# Evaluation of X-Linked Adrenoleukodystrophy Newborn Screening in North Carolina

**DOI:** 10.1001/jamanetworkopen.2019.20356

**Published:** 2020-01-31

**Authors:** Stacey Lee, Kristin Clinard, Sarah P. Young, Catherine W. Rehder, Zheng Fan, Ali S. Calikoglu, Deeksha S. Bali, Donald B. Bailey, Lisa M. Gehtland, David S. Millington, Hari S. Patel, Sara E. Beckloff, Scott J. Zimmerman, Cynthia M. Powell, Jennifer L. Taylor

**Affiliations:** 1 RTI International, Research Triangle Park, North Carolina; 2 Division of Genetics and Metabolism, University of North Carolina at Chapel Hill, Chapel Hill; 3 Department of Pediatrics, Duke University School of Medicine, Durham, North Carolina; 4 Department of Pathology, Duke University School of Medicine, Durham, North Carolina; 5 Department of Neurology, University of North Carolina at Chapel Hill, Chapel Hill; 6 Division of Pediatric Endocrinology, University of North Carolina at Chapel Hill, Chapel Hill; 7 North Carolina State Laboratory of Public Health, Raleigh; 8 Now at Division of Newborn and Childhood Screening, Maryland Department of Health, Laboratories Administration, Baltimore

## Abstract

**Importance:**

X-linked adrenoleukodystrophy (X-ALD) is a peroxisomal genetic disorder in which an accumulation of very long-chain fatty acids leads to inflammatory demyelination in the central nervous system and to adrenal cortex atrophy. In 2016, X-ALD was added to the US Recommended Uniform Screening Panel.

**Objective:**

To evaluate the performance of a single-tier newborn screening assay for X-ALD in North Carolina.

**Design, Setting, and Participants:**

This diagnostic screening study was of all newborn dried blood spot specimens received in the North Carolina State Laboratory of Public Health between January 2 and June 1, 2018, excluding specimens of insufficient quantity or quality. A total of 52 301 specimens were screened for X-ALD using negative ionization high-performance liquid chromatography tandem mass spectrometry to measure C24:0- and C26:0-lysophosphatidylcholine concentrations. Sanger sequencing of the adenosine triphosphate–binding cassette subfamily D member 1 (*ABCD1*) gene was performed on screen-positive specimens.

**Exposures:**

A medical and family history, newborn physical examination, sequencing of *ABCD1* on dried blood spot samples, and plasma analysis of very long-chain fatty acids were obtained for all infants with screen-positive results.

**Main Outcomes and Measures:**

The prevalence of X-ALD in North Carolina and the positive predictive value and false-positive rate for the first-tier assay were determined.

**Results:**

Of 52 301 infants tested (47.8% female, 50.6% male, and 1.7% other or unknown sex), 12 received screen-positive results. Of these 12 infants, 8 were confirmed with a genetic disorder: 3 male infants with X-ALD, 3 X-ALD–heterozygous female infants, 1 female infant with a peroxisome biogenesis disorder, and 1 female infant with Aicardi-Goutières syndrome. Four infants were initially classified as having false-positives results, including 3 female infants who were deemed unaffected and 1 male infant with indeterminate results on confirmatory testing. The positive predictive value for X-ALD or other genetic disorders for the first-tier assay was 67%, with a false-positive rate of 0.0057%.

**Conclusions and Relevance:**

This newborn screening pilot study reported results on 2 lysophosphatidylcholine analytes, identifying 3 male infants with X-ALD, 3 X-ALD–heterozygous female infants, and 3 infants with other disorders associated with increased very long-chain fatty acids. These results showed successful implementation in a public health program with minimal risk to the population. The findings will support other state laboratories planning to implement newborn screening for X-ALD and related disorders.

## Key Points

**Question:**

What is the analytical and clinical validity of a mass spectrometric method evaluating very long-chain fatty acyl-lysophosphatidylcholine species for the detection of X-linked adrenoleukodystrophy among newborns in North Carolina?

**Findings:**

In this newborn diagnostic screening study of 52 301 dried blood spot specimens, 3 male infants were confirmed to have X-linked adrenoleukodystrophy, 3 female infants were identified as heterozygous for X-linked adrenoleukodystrophy (carriers), 1 female infant had a peroxisome biogenesis disorder, 1 female infant had Aicardi-Goutières syndrome, 1 male infant received an indeterminate diagnosis, and 3 female infants had false-positive results.

**Meaning:**

The newborn screening results suggested that detecting increased concentrations of very long-chain fatty acyl-lysophosphatidylcholine species in dried blood spots is an effective method for identifying infants with X-linked adrenoleukodystrophy.

## Teaser

This diagnostic study evaluates the performance of a single-tier screening assay for X-linked adrenoleukodystrophy among newborns in North Carolina.

## Introduction

The X-linked adrenoleukodystrophy (X-ALD) disorder is a peroxisomal disorder caused by a deficiency of adenosine triphosphate–binding cassette transporter protein (adrenoleukodystrophy protein) encoded by the adenosine triphosphate–binding cassette subfamily D member 1 (*ABCD1*) gene (OMIM 300371).^1,2^ The protein transports very long-chain acyl-CoA esters into peroxisomes, the site of very long-chain fatty acid (VLCFA) beta-oxidation.^3^ In patients with X-ALD, VLCFAs accumulate in all tissues, primarily affecting the central nervous system and adrenal cortex. The 3 main phenotypes of X-ALD are Addison disease (adrenal insufficiency), adrenomyeloneuropathy, and cerebral adrenoleukodystrophy (CALD).^4^ Individuals with X-ALD are typically asymptomatic at birth; however, those with the severe phenotype—the childhood form of CALD—typically present between 2.5 and 10 years of age.^4^ Without treatment, patients with childhood CALD rapidly decline, and death typically occurs 2 to 4 years after onset of symptoms.^4^

Hematopoietic stem cell transplantation is the recommended treatment of patients with childhood CALD and is performed at the first sign of brain pathology as detected on magnetic resonance imaging (MRI).^4,5,6^ The MRI is rated using a Loes score, which indicates the severity of brain lesions.^7^ Hematopoietic stem cell transplantation can arrest the progression of cerebral demyelination if the patient is treated during an asymptomatic or early stage.^6,8^

Most male infants diagnosed as having X-ALD also have adrenal insufficiency and can experience adrenal function impairment as early as 6 months of age that may lead to morbidity and mortality.^9^ Therefore, it is important to identify infants as early as possible and to monitor their serum adrenocorticotropic hormone and cortisol levels in an effort to institute lifesaving hormone replacement therapy if abnormalities are observed.

The Advisory Committee on Heritable Disorders in Newborns and Children reviewed evidence demonstrating effective laboratory technologies available for X-ALD newborn screening (NBS) as well as the benefits of early identification and treatment on health outcomes. Based on its interpretation of the data, this advisory committee recommended the addition of X-ALD to the Recommended Uniform Screening Panel in August 2015, a move that the secretary of the US Department of Health and Human Services approved in February 2016. After X-ALD was added to the Recommended Uniform Screening Panel, 8 states added this disorder to their NBS panel before the pilot study in North Carolina began; since the pilot began, another 8 states and the District of Columbia have added it to their NBS panel.^10^ The North Carolina team—RTI International, the University of North Carolina at Chapel Hill (UNC-CH), Duke University, and the North Carolina State Laboratory of Public Health (NCSLPH)—implemented a statewide X-ALD pilot study using a testing method developed by the Centers for Disease Control and Prevention.^11^

The objectives of the present study were to evaluate the utility of screening for X-ALD using 2 biochemical analytes to support the identification of X-ALD and other peroxisomal disorders and reduce false-positive results, to adopt a follow-up plan that was feasible for the state of North Carolina, and to gauge the amount of additional testing needed for family members who had an infant diagnosed as having X-ALD through NBS.

## Methods

### Samples

In this diagnostic NBS study, X-ALD screening was performed prospectively between March 5 and July 31, 2018, using 52 301 identifiable consecutive specimens received at NCSLPH between January 2 and June 1, 2018. This report adhered to the Standards for Reporting of Diagnostic Accuracy (STARD) reporting guideline. The RTI and UNC-CH institutional review boards approved the design and methods and determined that this pilot study did not meet criteria for human subjects research; therefore, informed participant consent was waived.

### Laboratory Test

The quantification of 1-tetracosanoyl-sn-glycero-3-phosphocholine (C24:0- lysophosphatidylcholine [LPC]), in which C24:0 denotes a saturated fatty acid with a carbon chain length of 24, and 1-hexacosanoyl-2-hydroxy-sn-glycero-3-phosphocholine (C26:0)-LPC was conducted using a published method.^11^ In brief, a 3.2-mm-diameter circle from dried blood spot (DBS) specimens was extracted in 96-well plates with 100 μL of methanol containing the internal standard, 1-hexacosanoyl-d4-2-hydroxy-sn-glycero-3-phosphocholine (d_4_-C26:0-LPC). Plates were covered with an adhesive seal and shaken at 650 rpm for 30 minutes at 31 °C. Extracts were transferred to new polypropylene 96-well plates and heat-sealed with aluminum seals. The analysis was performed using high-performance liquid chromatography tandem mass spectrometry (HPLC-MS/MS) in negative ion mode on a Waters Acquity TQD LC/MS/MS system (Waters Corp) equipped with a 2777C sample manager and a Waters 1525μ Binary HPLC pump. Extracts (20 μL) were injected onto a 2.1 × 50-mm, 3.5-μm X-Terra MS C8 column, with a 2.1 × 5-mm 3.5-μm guard column (Waters Corp). Analytes were separated by isocratic elution using ammonium acetate, 5 mmol/L, in 50:50 (v:v) acetonitrile: methanol as the mobile phase, with a retention time of approximately 1 minute. Analytes were detected by selected reaction monitoring using the transitions m/z 592.50 to 367.35 (C24:0-LPC), m/z 620.5 to 395.4 (C26:0-LPC), and m/z 624.5 to 399.4 (d_4_-C26:0-LPC). Quantification of C26:0-LPC and C24:0-LPC was conducted by multiplying peak area ratios of C24:0-LPC or C26:0-LPC to d_4_-C26:0-LPC, with the d_4_-C26:0-LPC concentration in the extraction solvent (0.16 μmol/L) and the dilution factor of blood from a 3.2-mm DBS punch in 100 μL of extraction solvent. The overall run time per sample was approximately 3 minutes.

Sanger sequencing of the *ABCD1* gene was performed on all screen-positive samples at the Duke Clinical Molecular Diagnostics Laboratory. The coding sequences and flanking intronic sequences (minimum of 20 base pairs) of exons 1 through 10 of *ABCD1* were amplified using polymerase chain reaction from purified genomic DNA isolated from two 3.2-mm DBS punches. Bidirectional sequencing was performed, and the results compared with the reference DNA sequence (NM_000033.3). Variants were classified as pathogenic, likely pathogenic, variants of uncertain significance, likely benign, and benign according to the American College of Medical Genetics and Genomics/Association for Molecular Pathology criteria.^12^ Evidence used to classify variants included allele frequency from the Genome Aggregation Database of population variation, the ClinVar database of clinically curated variation, a locus-specific database at the ALD info website,^13^ previous reports in the scientific literature, and in silico prediction tools (eg, PolyPhen-2, MutationTaster).

### Screening Algorithm

**Figure 1.**
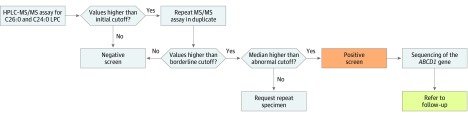
Screening Algorithm Used to Evaluate Newborn Dried Blood Spot Specimens A first-tier high-performance liquid chromatography tandem mass spectrometry (HPLC-MS/MS) assay in negative ion mode was used for the initial screening of specimens. All specimens that screened positive were sent for sequencing of the adenosine triphosphate–binding cassette subfamily D member 1 (*ABCD1*) gene, and the infant was referred to follow-up. Multiple specimens from the same infant with borderline results that were also classified as abnormal were sent for sequencing and referred to follow-up. C24 indicates 1-tetracosanoyl-sn-glycero-3-phosphocholine; C26, 1-hexacosanoyl-2-hydroxy-sn-glycero-3-phosphocholine; and LPC, lysophosphatidylcholine

Per the testing algorithm (Figure 1), specimens were initially tested using the first-tier HPLC-MS/MS assay. Cutoff values of 0.175 μmol/L for C24:0-LPC and 0.08 μmol/L for C26:0-LPC were used to retest samples in duplicate. The 3 values for C24:0-LPC and C26:0-LPC (from the initial plate and the 2 retest plates) were examined. If the median concentration for C26:0-LPC was at least 0.15 μmol/L or the C26:0-LPC concentration was between 0.08 μmol/L and 0.15 μmol/L and the C24:0-LPC concentration was 0.175 μmol/L or higher, then the specimen was classified as screen-positive. Specimens with a median C26:0-LPC concentration of 0.08 μmol/L or higher but lower than 0.15 μmol/L and a C24:0-LPC concentration lower than 0.175 μmol/L were classified as borderline, and an additional specimen was requested. Multiple specimens from the same infant with borderline results were classified as screen-positive.

### Follow-up of Screen-Positive Specimens

**Figure 2.**
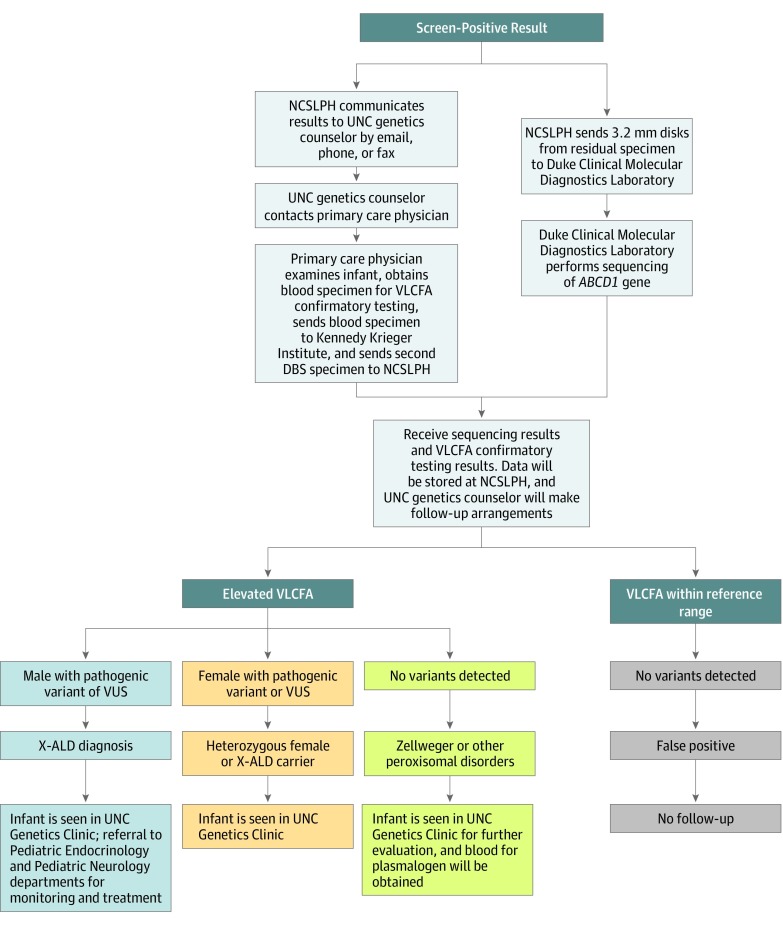
Follow-up Protocol for Screen-Positive Specimens The flowchart shows the protocol for confirmatory testing and short-term follow-up of screen-positive specimens through diagnosis. *ABCD1* indicates the adenosine triphosphate–binding cassette subfamily D member 1 gene; DBS, dried blood specimen; NCSLPH, North Carolina State Laboratory of Public Health; UNC, University of North Carolina; VLCFA, very long-chain fatty acid; VUS, variant of unknown significance; and X-ALD, X-linked adrenoleukodystrophy.

Information about screen-positive cases was referred to a genetic counselor at UNC-CH for follow-up. Subsequent patient care was provided according to the follow-up protocol presented in Figure 2.

### Statistical Analysis

Descriptive statistics, such as means and SDs, and graphical representations of the data were derived using Microsoft Excel (Microsoft Corp). Demographic categories were reported by frequency as well as by percentages. Positive predictive value was calculated by dividing the true-positive cases by the sum of the positive and negative cases. The false-positive rate was calculated by dividing the false-positive cases by the true-positive and true-negative cases.

## Results

### Specimen Testing Results

The demographic data found on the NBS card, including sex, birth weight, and age at infant at DBS collection, of all the specimens sent to the NCSLPH during the study period are provided in Table 1. The population was 47.8% female, 50.6% male, and 1.7% other or unknow sex. The mean (SD) concentrations in this population were 0.080 (0.036) μmol/L for C24:0-LPC and 0.033 (0.019) μmol/L for C26:0-LPC. Of 52 301 specimens, 833 (1.6%) had screening results above the initial cutoff and were retested. Based on the initial and retest results of the original DBS specimens, 11 cases were classified as abnormal and referred for follow-up. Another 45 specimens had a borderline result (16 female and 29 male infants), of which repeat specimens were received in 27 cases (60%), and no response or a declination in 18 cases. Of the repeat specimens, 25 of 27 (93%) had a normal test result, 1 was of insufficient quality to be tested, and 1 had a second borderline test result. This latter case was classified as screen-positive and referred for follow-up, totaling the screen-positive cases to 12.

**Table 1.** North Carolina Overall Study Population Demographics

Characteristic	No. (%) of Participants
Sex	
Female	25 026 (47.8)
Male	26 443 (50.6)
Other/unknown	880 (1.7)
Birth weight	
Normal (>2500 g)	46 174 (88.3)
Low (2499-1501 g)	4447 (8.5)
Very low (1500-1000 g)	888 (1.7)
Extremely low (<1000 g)	840 (1.6)
Age at DBS collection, h	
<24	832 (1.6)
24-48	43 400 (83.0)
49-72	3111 (5.9)
73-96	388 (0.7)
97-168	505 (1.0)
>168	4112 (7.9)

Abbreviation: DBS, dried blood spot.

**Figure 3.**
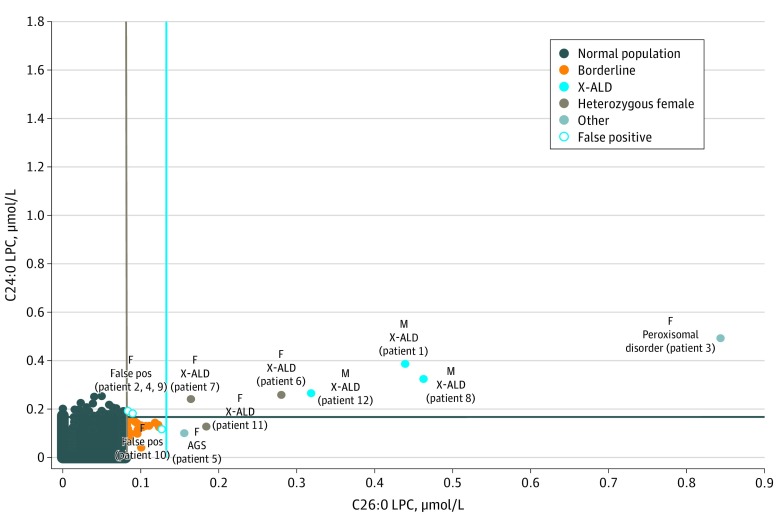
Two-Dimensional Plot of the C24:0-Lysophosphatidylcholine (C24:0-LPC) Analyte vs the C26:0-LPC Analyte Screen-positive cases are as follows: X-linked adrenoleukodystrophy (X-ALD) hemizygotes, false-positive (False pos), X-ALD heterozygous female, and other disorders. The cyan vertical line is set at 0.15 μmol/L, and the navy horizontal line is set at 0.175 μmol/L to capture all the samples with increased concentrations of C26:0-LPC and C24:0-LPC, respectively. The tan vertical line at 0.08 μmol/L represents the borderline cutoff value for C26:0-LPC. The patient numbers in parentheses correspond to those given in Table 2. AGS indicates Aicardi-Goutières syndrome; F, female; and M, male.

Figure 3 is a 2-dimensional plot of the C24:0-LPC vs C26:0-LPC analytes. Six of 12 screen-positive specimens (50%) had increased concentrations of both analytes and included all 3 male infants diagnosed as having X-ALD, 2 female infants diagnosed as heterozygous for X-ALD, and 1 female infant diagnosed as having a peroxisomal biogenesis disorder. An additional 2 infants (17%)—a female heterozygous for X-ALD and a female infant with Aicardi-Goutières syndrome—had increased C26:0-LPC concentrations only. Three specimens with borderline C26:0-LPC concentrations and increased C24:0-LPC concentrations included 2 female infants who were false-positive and 1 male infant with indeterminate VLCFA results and a variant of unknown significance (VUS) in *ABCD1*. None of the false-positive specimens had increased concentrations of C26:0-LPC, and only 1 case had consistent borderline C26:0-LPC concentration and C24:0-LPC concentration results within the reference range.

### Confirmatory Testing and Short-term Follow-up Results

Table 2 provides VLCFA and sequencing results for the 12 screen-positive cases, as well as final diagnoses. Gene sequencing identified *ABCD1* variants in 6 of 12 screen-positive specimens. Three of these variants—all observed in hemizygous male infants—were classified as VUSs per American College of Medical Genetics and Genomics/Association for Molecular Pathology criteria.^14^ For the VUSs reported in patients 1 and 8, some evidence suggesting pathogenicity was available, including publications citing different missense changes at the same amino acid positions in individuals affected with X-ALD.^15,16^ The third VUS, observed in patient 4, had an allele frequency greater than expected for a pathogenic variant (23 of 12 877 alleles in the African population, including 7 hemizygous male infants in the Genome Aggregation Database),^17^ suggesting that the VUS could be benign. The remaining variants, observed in 2 female infants heterozygous for X-ALD and 1 male hemizygous for *ABCD1*, were pathogenic or likely pathogenic. After initial gene sequencing failed to detect a variant in *ABCD1*, 1 female infant was later found to have a heterozygous deletion in *ABCD1* through additional molecular confirmatory testing. No *ABCD1* variants were detected in the remaining 6 screen-positive samples.

**Table 2.** Plasma Very Long-Chain Fatty Acid Confirmatory Test Results for Screen-Positive Cases

Patient No.	Sex	C26:0, μg/mL	C26:1, μg/mL	C24:0, μg/mL	C22:0, μg/mL^a^	C26/C22^b^	C24/C22^b^	VLCFA Diagnostic Interpretation	Molecular Testing Result	Final Diagnosis
1	M	1.25	0.76	32.48	15.8	0.079	2.05	Increased C26:0, C26:1, C24:0, C24/22, C26/22	1 Hemizygous VUS in *ABCD1*: c.1522C>T; p.Pro508Ser	X-ALD
8	M	0.95	0.43	31.19	20.6	0.046	1.51	Increased C26:0, C26:1, C24:0, C24/22, C26/22	1 Hemizygous VUS in *ABCD1*: c.631C>T; p.Leu211Phe	X-ALD
12	M	1.18	0.58	29.13	17.9	0.066	1.63	Increased C26:0, C26:1, C24:0, C24/22, C26/22	1 Hemizygous likely pathogenic in *ABCD1*: c.1772G>A; p.Arg591Gln	X-ALD
6	F	0.38	0.57	7.76	6.7	0.057	1.16	Increased C26:1, C24/22, C26/22; low C22:0	1 Heterozygous pathogenic in *ABCD1*: c.1661G>A; p.Arg554His	X-ALD heterozygous female
7	F	0.89	0.64	27.68	18.0	0.050	1.54	Increased C26:0, C26:1, C24/22, C26/22	1 Heterozygous pathogenic in *ABCD1*: c.1895C>T; p.Thr632Ile	X-ALD heterozygous female
11	F	1.12	0.84	41.37	35.5	0.032	1.17	Increased C26:0, C26:1, C24:0, C24/22, C26/22	Heterozygous deletion exon 7-10 in *ABCD1*^c^	X-ALD heterozygous female
3	F	2.89	2.19	14.90	8.8	0.330	1.70	Increased C26:0, C26:1, C24/22, C26/22	No *ABCD1* variants detected; compound heterozygous for pathogenic variants in *PEX1* gene^c^	Peroxisomal biogenesis disorder
5	F	NA^d^	NA^d^	NA^d^	NA^d^	NA^d^	NA^d^	NA^d^	No *ABCD1* variants detected; homozygous pathogenic variant in *TREX1*^d^	Aicardi-Goutières syndrome
2	F	0.29	0.23	21.11	24.8	0.012	0.85	Normal	No *ABCD1* variants detected	False-positive
9	F	0.19	0.17	17.00	19.9	0.010	0.85	Normal	No *ABCD1* variants detected	False-positive
10	F	0.34	0.30	16.12	19.1	0.018	0.84	Normal	No *ABCD1* variants detected	False-positive
4	M	0.27	0.22	23.93	20.5	0.013	1.17	Slightly increased C24/C22	1 Hemizygous VUS in *ABCD1*: c.895C>T; p.His299Tyr	Indeterminate
Reference range^e^		0.05-0.41	0.05-0.36	6.87-28.31	8.43-33.51	0.002-0.018	0.64-1.04			

Abbreviations: *ABCD1,* ATP binding cassette subfamily D member 1; C22:0, docosanoic acid; C24:0, tetracosanoic acid; C26:0, hexacosanoic acid; C26:1, 17-hexacosenoic acid; F, female; M, male; NA, not available; *PEX1*, peroxisomal biogenesis factor 1; *TREX1,* 3 prime repair exonuclease 1; VLCFA, very long-chain fatty acid; VUS, variant of unknown significance; X-ALD, X-linked adrenoleukodystrophy.

SI conversion factors: To convert micrograms per milliliter to micromoles per liter, multiply by 2.52 for C26:0; by 2.53 for C26:1; by 2.71 for C24:0; by 2.94 for C22:0; by 0.86 for C26:0/C22:0; and by 0.92 for C24:0/C22:0.

^a^ Data to more than 1 decimal place were unavailable.

^b^ C26/C22 corresponds to C26:0/C22:0, and C24/C22 corresponds to C24:0/C22:0.

^c^ Sequencing results were determined from the peroxisomal disorders panel, not from a dried blood spot.

^d^ This infant was cared for at another facility; the VLCFAs testing was performed at a different laboratory, and a next-generation sequencing panel, rather than a dried blood spot, determined sequencing results.

^e^ Based on reports provided by the referral laboratory; reference range limits are the mean values plus or minus 2 SDs, as defined by the referral laboratory.

Three male infants were confirmed to have X-ALD, and all received follow-up visits with members of the genetics, endocrinology, and neurology departments at UNC-CH. The VLCFA analysis revealed increased C26:0, C26:1, and C24:0 concentrations and an increase in the ratios of C24:0 to C22:0 and of C26:0 to C22:0 in all cases. Two infants had examinations with results that were within references ranges (patients 8 and 12), but 1 had poor weight gain and brisk reflexes (patient 1). All had serum electrolyte and cortisol levels within reference ranges, but 2 infants had increased adrenocorticotropic hormone levels at 6 months (patient 8) and 9 months (patient 12) of age. To date, those 3 infants remain asymptomatic for neurological involvement. All 3 families reported no known family history of X-ALD. However, 1 family (patient 8) described nonspecific findings, including attention-deficit/hyperactivity disorder and learning difficulties, in a maternal uncle, although no diagnosis could be inferred from the information provided. Carrier testing was offered to the patients’ mothers through genetic testing and to brothers by using VLCFA testing. Only 2 families pursued recommended testing during the pilot study.

Three female infants were diagnosed as heterozygous for X-ALD (carriers), and all families received genetic counseling. The VLCFA analysis showed increased concentrations of C26:1 and increased ratios of C24:0 to C22:0 and of C26:0 to C22:0 in all cases. Two families had no additional children, no families had a reported history of X-ALD, and all parents declined carrier testing. One infant (patient 11) was seen at the UNC-CH Genetics Clinic because of increased VLCFA concentrations, and initially no variants were detected in the *ABCD1* gene. The plasmalogens and liver function results were within reference ranges, and a peroxisomal gene panel revealed a heterozygous partial deletion of exons 7 through 10 of *ABCD1*.

One female infant was diagnosed as having a peroxisomal biogenesis disorder. The VLCFA analysis showed the highest increases of C26:0 and C26:1 concentrations and ratio of C24:0 to C22:0 as well as an increased ratio of C26:0 to C22:0. On examination, this child had hypotonia, developmental delay, feeding problems, and abnormal eye movements. Additional testing revealed low plasmalogen to fatty acid ratios and compound heterozygous pathogenic variants in the peroxisomal biogenesis factor 1 (*PEX1*) gene.

One female infant had Aicardi-Goutières syndrome; she was under the care of another genetics center and did not undergo the confirmatory testing protocol specified by this pilot study. The primary care physician reported that this patient had microcephaly, ventricular septal defect, possible cortical malformations on MRI, bilateral hearing loss, and intra-abdominal calcifications without dysmorphic features on physical examination. Homozygous pathogenic variants in the 3 prime repair exonuclease 1 (*TREX1*) gene were found through whole-exome sequencing.

One male infant had a slight increase in the C24:0 to C22:0 ratio (patient 4), which was interpreted by the diagnostic laboratory as possible liver dysfunction. The patient was examined at 6 months of age and had prolonged neonatal hyperbilirubinemia but no evidence of liver dysfunction; the liver function test results were within reference ranges. An isolated increase in the C24:0 to C22:0 ratio is not consistent with a diagnosis of X-ALD or of a peroxisomal biogenesis disorder; thus, this case is likely a false-positive. However, the parents were advised to return in 2 years for reevaluation.

Three infants, all female, were categorized as having false-positive screening results, with no pathogenic variants, likely pathogenic variants, or VUS detected in *ABCD1* and normal levels of VLCFAs (patients 2, 9, and 10). One infant (patient 10) had a slight increase in the C26:0 to C22:0 ratio that was not considered significant. These patients had no further follow-up.

### Performance of Screening Assay

The newborn screening study identified 3 male infants with X-ALD and 3 female infants heterozygous for X-ALD. The incidence was estimated to be 1 in 8717 births, a slightly higher frequency than that observed by the New York NBS program^18^ but lower than the frequency reported by the Minnesota NBS program.^19^ The positive predictive value for the first-tier assay was 67%, and the false-positive rate, which was defined as cases sent to follow-up and diagnostic testing indicating that the infant did not have X-ALD or a related disorder, was 0.0057%. Only 1 borderline case was included in the false-positive rate. All other borderline cases were not sent to follow-up and were not included in this calculation.

## Discussion

### Evaluation of the Screening Algorithm

This pilot newborn screening study used the HPLC-MS/MS method in negative ion mode because it is more selective than alternative published methods; and the initial screening algorithm did not require adjustment during the course of the study. However, the build-up of blood spot matrix in the instrumentation caused a revision in maintenance procedures, including frequent replacement of the analytical and guard columns and mass spectrometer consumable parts. Alternative methods for X-ALD screening combines the analysis of C26:0-LPC with amino acid and acylcarnitine^20^ or lysosomal enzyme analysis^21^ and detects C26:0-LPC in the positive ion mode. These combined methods use flow injection analysis and are thus more suitable as a first-tier screening test. However, in positive ion mode, an isobaric interference increases the false-positive rate of these flow injection analysis methods, necessitating the use of a more specific second-tier test, such as the negative-ion HPLC-MS/MS method used in our pilot study.

Of the 45 borderline results, an additional specimen was received for 27 infants, and most of the results from the second specimen were normal. The borderline cutoff value was set conservatively to ensure that no infants with X-ALD were missed, but the evidence from this study suggested that the specimens with borderline results were at a low risk for X-ALD. In the future, with more population screening data, it may be possible to eliminate this category.

The present study also found that the screening target, male infants with X-ALD, showed increases in both analytes, suggesting that measurement of C24:0-LPC in addition to C26:0-LPC is valuable in discerning specimens from patients with X-ALD or other peroxisomal disorders, but this additional analyte should not be considered the primary marker. At the time of the study, no stable isotope-labeled internal standard was available for C24:0-LPC; however, d_4_-C24:0-LPC is now commercially available, and additional studies are needed to evaluate this internal standard on the accuracy and precision of C24:0-LPC quantification.

### Short-term and Long-term Follow-up

The follow-up protocol included a thorough family history, and carrier testing was offered to mothers because of the reproductive risks and because 80% of female infants develop signs of neurological dysfunction by 60 years of age.^22^ Male siblings were also offered testing because there is a 50% risk that these male siblings will have X-ALD if their mother is a carrier. The parents of female carriers and 1 mother of a male infant with X-ALD did not pursue testing for themselves. The families may not have understood the full implications of the diagnosis despite receiving information, were not planning for more children, or were dissuaded by the cost of testing (not included in the pilot study) or lack of insurance. Additional materials may need to be developed to help families understand the various effects X-ALD may have and how to share information with their extended family.

Long-term follow-up is an important component of a complete NBS system and is particularly important for newborns identified with X-ALD. In total, 40% to 45% of male infants with X-ALD have no symptoms until adulthood.^4^ There is no published correlation between genotype and phenotype or age at onset;^23,24,25^ therefore, routine clinical monitoring is critical for the initiation of timely and effective interventions and for assessing the clinical course of cases identified through NBS. The New York State NBS Program published recommendations for follow-up of presymptomatic boys in childhood.^26^ These recommendations have been adopted by most states screening for X-ALD and this pilot study. The adapted protocol used to follow-up with patients identified in this study includes monitoring of adrenal function with tests for serum adrenocorticotropic hormone and cortisol every 6 months until 18 years of age and then annually, and a neurology evaluation, including brain MRI without contrast annually, up to 3 years of age, every 6 months from 3 to 10 years of age, and annually from 11 to 18 years of age. Additional brain MRI may be added outside the proposed time frame if there are clinical concerns because of history or neurologic examination findings.

In conjunction with follow-up recommendations for presymptomatic male infants, clinical referral networks with expertise in medical genetics, neurology, endocrinology, and the provision of hematopoietic stem cell transplantation need to be established to coordinate follow-up for individuals with presymptomatic X-ALD.^27^ Coordinating follow-up care with 3 subspecialties may be challenging for some states because of the lack of available specialists and funding; however, the coordination will facilitate more effective care and proper surveillance of these patients. These centers could potentially be used to track long-term outcomes of patients, which are critical to understanding which subtypes of X-ALD are identified through NBS and whether early identification and treatment of childhood CALD is successful. It will also provide insight into the natural progression of adrenal and neurologic involvement and allow refinement of screening and follow-up protocols to include guidance on infants who have disorders other than X-ALD.

### Limitations

The testing method is intended for screening and should be accompanied by diagnostic testing and medical evaluation. This method cannot predict disease severity, and long-term follow-up data were not collected to evaluate long-term outcomes of identified infants.

## Conclusions

The North Carolina pilot study implemented a screening program that detected true screen-positive specimens with a high degree of analytical specificity and identified 3 male and 3 female newborns with X-ALD as well as other disorders. Future work is needed to evaluate the long-term data on patients identified with X-ALD through NBS to understand the clinical presentation, course of the condition, effectiveness of early treatment, ability of the health care system to provide follow-up care, and effect on families.
